# Coating of Flexible PDMS Substrates through Matrix-Assisted Pulsed Laser Evaporation (MAPLE) with a New-Concept Biocompatible Graphenic Material

**DOI:** 10.3390/nano12203663

**Published:** 2022-10-18

**Authors:** Michela Alfe, Giuseppina Minopoli, Massimiliano Tartaglia, Valentina Gargiulo, Ugo Caruso, Giovanni Piero Pepe, Giovanni Ausanio

**Affiliations:** 1 Institute of Sciences and Technologies for Sustainable Energy and Mobility (CNR-STEMS), P.le V. Tecchio 80, 80125 Naples, Italy; 2 Department of Molecular Medicine and Medical Biotechnology, University of Naples Federico II, via Pansini, 5, 80131 Naples, Italy; 3 Department of Chemical Sciences, University of Naples Federico II, via Cinthia 4, 80126 Naples, Italy; 4 Department of Physics “E. Pancini”, University of Naples Federico II, via Cinthia 4, 80126 Naples, Italy

**Keywords:** graphene related materials, thin films, biocompatibility, MAPLE, laser deposition

## Abstract

In this study, matrix-assisted pulsed laser evaporation (MAPLE) was used to deposit graphene-like materials (GL), a new class of biocompatible graphene-related materials (GRMs) obtained from a controlled top-down demolition of a carbon black, on silicone slices to test their potential use as functional coating on invasive medical devices as indwelling urinary catheters. Results indicate that the relevant chemical-physical features of the deposit (controlled by FTIR and AFM) were maintained after MAPLE deposition. After deposition, GL films underwent a biological survey toward target cellular lines (murine fibroblast NIH3T3, human keratinocytes HaCAT and the human cervical adenocarcinoma epithelial-like HeLa). Results indicate that the GL films did not lead to any perturbations in the different biological parameters evaluated. The presented results and the possibility to further functionalize the GL or combine them with other functional materials in a hybrid fashion to assure a tighter adhesion onto the substrate for use in harsh conditions open the door to practical applications of these new-concept medical devices (drug delivery, next generation flexible devices, multifunctional coatings) paving the way to the prevention of nosocomial infections driven by catheterization through antibiotics-free approaches.

## 1. Introduction

The health emergency linked to the pandemic outbreak has posed the critical issue of long-term patients’ hospitalization in intensive care units (ICUs) requiring the use of invasive medical devices. Intravascular catheters are indispensable in current medical practice as well as urinary catheters, although their use exposes patients to risk of healthcare-associated infections (HAI) as local and systemic infectious complications (for example bloodstream infections, BSI) and catheter-associated urinary tract infections (CAUTIs) which have significant morbidity and mortality [1]. The resistance of microbial communities toward antibiotics pushes towards antibiotics-free approaches. Moreover, the ability of the microbial pathogens to form biofilms stuck on the surface of the catheter is also of great concern [2]. In an effort to prevent HAI, catheter modifications at the surface have been proposed: (i) coating with materials characterized by anti-fouling properties (hydrogels, polymers, polysaccharides such as hyaluronic acid and heparin, polyzwitterionic polymers, enzymes), polysaccharides from marine sources (alginate, ulvan, agarose, carrageenan) or cyanobacterial polymer-based materials [3,4,5]; (ii) release-based approaches using antibiotics, antiseptics, nitric oxide, 5-fluorouracil, metal nanoparticles and polymer/nanoparticles hybrid systems [3,4,5,6,7,8]; and (iii) contact-killing approaches employing quaternary ammonium compounds, chitosan, antimicrobial peptides, and enzymes [4,5]. Each of these methodologies showed benefits and limitations, for example polymer-based coatings exhibit short-term durability, and even if the release-based strategies persist as a cost-effective approach, metal nanoparticles (apart from Ag ion-releasing coatings in the form of Ag coatings, Ag alloy with gold, palladium, Ag-containing polymers and Ag nanoparticles [6] since silver is one of the few antimicrobial agents for urinary catheter coatings approved by the U.S. Food and Drug Administration [6]) can be toxic for non-targeted organism, and antibiotic-based coatings can contribute to increased bacterial antibiotic resistance [5,6].

Carbon-based and in particular graphene-based materials are also emerging as possible coatings for catheters and biomedical devices [9,10]. The studies on these coatings are still in their infancy and a comprehensive picture of their potential application cannot be drawn due to the great variability in the results and approaches and also due to the lack of investigation on the in vivo utilization of such graphene-based antimicrobial surfaces to reveal the actual in vivo antimicrobial performance and biocompatibility.

The control of coating thickness and film uniformity is a relevant general issue related to the surface coating. Former items, indeed, represent key elements to determine the overall properties of innovative materials and for circumventing issues that do not allow the development of new solutions for various research fields. For example, single layer materials growth requires substrates having an atomically flat surface properly treated [11,12] as reported for the development of devices where the transport properties are heavily influenced by the used deposition techniques expected to be compatible with the entire nanofabrication protocol [13,14,15,16] or of electronic devices [17,18].

To date, there are many options to finely tune the surface chemistry and the overall characteristics of the catheter coating including chemical deposition, dip-coating, spin-coating, photo–chemical deposition, physical vapor deposition and sputtering deposition techniques [19]. The major drawback of these methods is the scarce adherence of the coating mainly due to the use of solvents. To achieve a fine control of the coating and a well adherent film, one of the best options could be the matrix-assisted pulsed laser evaporation (MAPLE) technique. MAPLE is a solvent-free technique particularly suitable for obtaining homogeneous, ultra-thin, well adherent coatings over any desired substrate by maintaining the chemical structure and the physiochemical properties of the deposited material. MAPLE is a top-notch choice to coat non planar-shaped substrates with delicate organic molecules including polymers, enzyme, proteins, and drugs [20,21] preserving their characteristics and activity. MAPLE assures an excellent control over thickness, adhesion and homogeneity of the deposited film [4,21] and exhibits many benefits over conventional methods (drop casting, dip-coating, spin coating, Langmuir–Blodgett technique) for manufacturing multifunctional coatings on flexible supports, including medical devices. Furthermore, since MAPLE ensures an optimal coating adhesion on the biomedical surface, a better control over antibacterial properties can be achieved. The resulting advantages are also related to the long-term stability of the coated surface. MAPLE is particularly suited for the deposition of organic molecules, polymers and biomolecules [22,23,24,25,26], but it also allows for the fabrication of either inorganic, organic, or hybrid coatings with a high versatility [27,28,29,30]. Recently, MAPLE techniques have been used also for the preparation of coatings and layered materials based on carbon-based nanomaterials including carbon nanotubes (CNTs) and graphene-related materials (GRMs) [30,31,32].

In the last few years, advanced carbon-based materials found applications in many technological fields [33,34,35], but recently they are emerging as possible antimicrobial coatings for catheters [36]. Graphene-related materials (GRMs) have recently attracted tremendous interest in medical application thanks to their chemical-physical tuneability [37]. Biological effects, mechanisms of the action and toxicity exerted by GRMs are almost unexplored and a clear categorization of the structure-activity relationships (SARs) is still far from being achieved. So far, only few publications on the biological effects of GRMs on living cells have been published [38,39,40]. In addition, the wide variability of GRMs in terms of structure, composition and properties (even within the same GRM category) opens the door to controversial biological effects in both in vitro and in vivo contexts [41].

GRMs including few-layer and multi-layered structures can be produced from C-based sources different from graphite such as carbon black, coke, carbonized/pyrolyzed wastes and biomasses through many approaches [42,43,44,45,46,47,48,49,50]. Graphene-like materials from coke have been produced implementing different approaches, but in all the cases, few-layered graphenic nanosheets with characteristics similar to graphite-derived ones (comparable graphenic sheets size) have been obtained. Sierra and coworkers [43,44,45] produced graphene-like materials by coke oxidation and further exfoliation by ultrasounds and also by a mechanochemical approach (one hour milling by using a 2:1 wt.% mixture of stearic acid and coke). Saha and coworkers proposed the production of graphene-like materials from petroleum coke by electrochemical exfoliation after an oxidative treatment [42].

Due to the high carbon content, plastic wastes can be considered a feedstock of choice for the production of graphene-like materials. Monolayer, few-layer and multi-layer graphene (including flash graphene) have been obtained from plastic wastes through many synthetic approaches [46]: (i) thermal decomposition (pyrolysis) of the plastics directly over a metal substrate or in the presence of a catalyst; (ii) chemical vapor deposition (CVD) on a metal substrate using hydrocarbons evolving from the thermal decomposition of the plastics; (iii) thermal decomposition followed by ball milling and microwave sintering; and (iv) flash Joule heating (FJH). Graphene-like materials have been produced also starting from bio-precursors and waste biomasses [47,48,49,50]. Bio-precursors as glucose, xylitol, sucrose, caramel, chitosan, alginate, white straw, palm oil and others allowed for the production of graphene-like materials by classical thermal methods (pyrolysis, carbonization, graphitization), chemical treatments (chemical activation), hydrothermal methods, template-based confinement method and combined approaches [49]. The quality and the characteristics of biomass-derived graphene-like materials are highly dependent on the precursor and the manufacturing technique. Graphene-like materials obtained from a controlled top-down demolition of a nanostructured carbon black (CB) in which they are embedded [51,52] are nanometric sized graphene stacked layers and exhibit peculiar chemical-physical properties and promising bio-safety (differently from the most GRMs) [40,53,54,55,56], forecasting feasible applications in many fields including bioelectronics, sensing and functional coatings. The synthetic approach offers control of the product’s relevant chemical-physical features, and it is suitable for bulk production. As concerns their promising bio-safe properties, CB-derived graphene-like materials (hereinafter GL) exhibit antimicrobial activity toward living cells through a bacteriostatic action [55]. The presence of GL in the of *S. aureus* cells culture media, one of the most prevalent Gram-positive bacteria associated with CAUTIs, induces a severe decrease of colony forming units [55]. Most interestingly, there is no evidence of *S. aureus* cell membrane leakage caused by GL, but the instauration of an intimate contact between the cells and the GL makes the material able to act as a bacteriostatic agent more than a cells toxic agent. In addition, the toxicological profile of GL has been assessed in vivo using embryonic zebrafish (*Danio rerio*) indicating their good biocompatibility on a vertebrate model [40]. 

In this work, MAPLE was used to deposit GL nanoparticles on silicone slices to test their potential use as functional coating on invasive medical devices as indwelling urinary catheters. PDMS elastomer was used due to its reasonable cost, rapid and customisable manufacturing, and ease of use. Among the wide PDMS combinations, the Sylgard 184 elastomer was selected being the most commonly used in biological-based research. The PDMS is a hydrophobic material and a reliable coating of molecules, and particles from water suspensions with conventional approaches is not feasible (Supporting Information, Figure S1). MAPLE approach is a leading technique able to overcome this limitation. Thanks to the characteristic absorption in the visible region of the GL [51,54], a variant of MAPLE method has been exploited, namely inverse matrix-assisted pulsed laser evaporation (IMAPLE). In this context, the GL act as a “self-matrix” thus allowing an efficient desorption of the frozen suspension in water without the use of light-absorbing solvents (acting as matrix in the laser-assisted desorption phenomena). 

An important parameter concerning the MAPLE deposition is the wavelength (or photon energy) of the laser light used for target ablation/evaporation. The laser light wavelength must be absorbed by matrix (most common solvents are almost transparent to IR radiation while they absorb UV radiation) but at the same time damages of local absorbers must be avoided. In our specific experimental condition (Nd:YAG laser used at NIR wavelength λ = 1064 nm) the optical characteristics of GL (broad band absorption extending to NIR region) allow the triggering of the target ablation at lower energy per photon (Ep = 1.17 eV) compared with Mid-UV light (Ep = 4.13–6.20 eV for λ = 200–400 nm) by keeping the same laser fluence (F) thus limiting the occurrence of GL modification phenomena (e.g., thermal carbonization).

The advantage in using GL, apart from the optical properties and well-established biocompatibility, also lies in the possibility to achieve stable colloidal suspensions in water [52], hardly obtainable with CB unless using surfactants.

Before and after deposition, the GL in water suspension were inspected by a biological survey toward target cellular lines (murine fibroblast NIH3T3, human keratinocytes HaCAT and the human cervical adenocarcinoma epithelial-like HeLa) to test for any perturbations in the different biological parameters evaluated, including the cytotoxic potential. Although in vitro and in vivo surveys have been already conducted on GL water suspensions on specific cells lines and vertebrate models [40,54,55], it has been widely demonstrated that the cellular response is cell line-dependent [57] and this outlines the importance of studying multiple cell lines for specific biological applications.

## 2. Materials and Methods

### 2.1. Materials

All chemicals were purchased from Merck KGaA, Darmstadt, Germany (ACS grade) and used without further purification. The carbon black (CB) used for the GL production (furnace type, CB N110, according to the ASTM classification) was provided by Sid Richardson Carbon Co. (Fort Worth, TX, USA).

### 2.2. Instrumental Methods

FTIR spectra in the 400–4000 cm^−1^ range were acquired on a Perkin-Elmer MIR spectrophotometer in transmission mode. The spectra were acquired on KBr pellets, with a resolution of 2 cm by collecting 8 scans and correcting the background noise. 

The size and shape of drop-casted and MAPLE-deposited films were examined by an atomic force microscope (AFM Digital Instruments Nanoscope IIIa, Bruker, MA, USA) equipped with a sharpened silicon tip with a radius of less than 10 nm. The images of the surface profiles were obtained by operating the microscope in the tapping mode, with a scan size and rate of 2 μm and 1 Hz, respectively. The thickness of the GL films on PDMS slices was evaluated by AFM on several edges of the deposited film and it was 200 nm ± 100 nm.

### 2.3. Preparation of the PDMS Substrates and GL Suspension for MAPLE Deposition

Polydimethylsiloxane (PDMS) Sylgard 184 elastomer slices were prepared by mixing a 10:1 ratio of elastomer to curing agent, degassing, and baking for 24 h at 60 °C. Before curing, the elastomer was poured into a standard 60 mm × 15 mm polystyrene Petri dish acting as a flat stamp (around 2 mL in each stamp). The thickness of the slides was measured with a caliper and was around 1–2 mm.

GL nanoparticles were obtained by performing a top-down demolition of a nanostructured carbon black in accordance with the approach reported elsewhere [51] and here reported in brief: CB powder (500 mg) was treated under stirring and reflux for 90 h with 10 mL of concentrated nitric acid (HNO_3_, 67%) at 100 °C. The oxidized carbonaceous material (hereinafter GL-ox) was then recovered by centrifugation and further purified from residual acid traces by several washings with distilled water and in the end dried at 105 °C. An amount of GL-ox was suspended in distilled water at a mass concentration of 1 mg/mL and treated with hydrazine hydrate (35 μL of hydrazine hydrate for each mg of GL-ox) at 100 °C under stirring and reflux for 24 h. After that, the hydrazine excess was neutralized by diluted nitric acid (4 M) and the GL were recovered by centrifugation as a black solid. GL were at first purified from reactants traces by two water washings followed by centrifugation and then by under-vacuum filtration on Durapore^®^ Membrane Filter, 0.22 µm. The GL, recovered on the filter, were diluted with distilled water establishing a concentration of 1 wt.% and stored. The GL suspension remains stable and no phase separation is visible. To assure concentration homogeneity before each deposition, the GL suspension underwent ultrasonic agitation before use. For preliminary cytotoxicity potential assessment, the 1 wt.% GL suspension was diluted to 1 mg/mL with sterile water and used as the stock solution for further dilutions (from 50 to 0.1 μg/mL).

### 2.4. MAPLE Deposition

**Figure 1:**
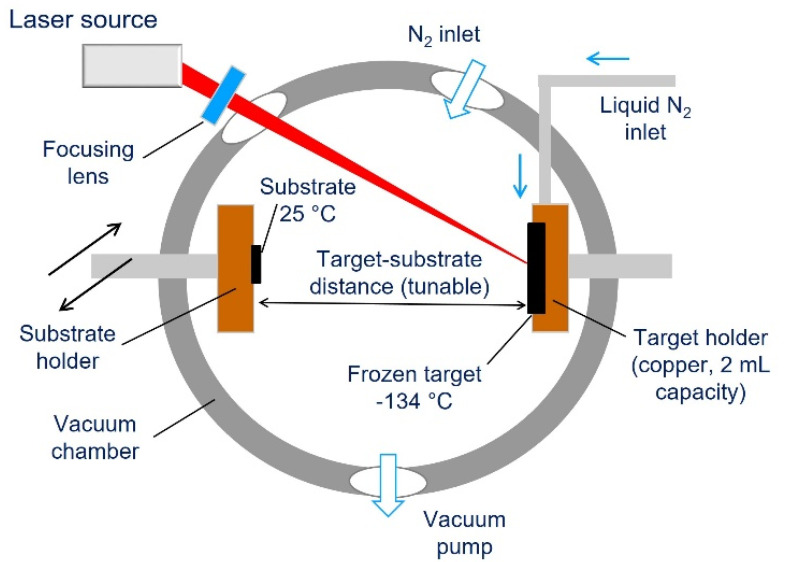
Schematic representation of the deposition system. The substrate holder can be moved to tune the inter distance between target and substrate (black arrows).

A MAPLE set-up equipped with a Nd:YAG 1064 nm laser and two pumps (a rotary pump for bootstrap and a turbomolecular pump for high vacuum) was used. The MAPLE apparatus configuration is reported in Figure 1. The target holder is a copper vessel of 2 mL capacity. The copper vessel was previously treated with glacial acetic acid to eliminate the surface native oxide. The GL suspension was used at the concentration of 1 wt.% (0.01 g/mL) in water. This concentration value was selected after an optimization study and allowed obtaining homogeneous liquid dispersions with the highest possible concentration of GL. The GL water suspensions were sonicated for 15 min at room temperature to assure completely homogeneous dispersions. 

The GL suspension was poured in the copper holder (MAPLE target) and quickly pre-frozen by immersing the holder in liquid nitrogen. The temperature at the MAPLE target, measured by a thermocouple was about −133 ± 10 °C. Once the GL suspension (target) was frozen, the target was placed into the vacuum chamber in thermal contact with a liquid nitrogen reservoir and evacuated. The Nd:YAG pulsed laser was operated at its fundamental wavelength (1064 nm). 

The laser beam reached the target at 45° and was partially focused to an ellipsoidal area of about 1.0 mm × 1.4 mm. The target was continuously moved with a 2D translation system in order to avoid overheating or holder drilling (possibly implying Cu contamination of the substrate). The laser beam scanned a target area of about 1.5 cm^2^. The pressure inside the chamber was kept constant (~10^−6^ torr) to assure reproducibility among the depositions and the target temperature. The inter distance between target and substrate was 8 mm. The following laser parameters were adopted: pulse repetition rate 4 Hz, pulse duration 7 ns, laser pulse energy 350 mJ/pulse (corresponding to a fluence of F = 25 J/cm^2^). The laser power was set above the solution ablation threshold (that was 120 mJ/pulse and F = 10 J/cm^2^, as determined from the increase in pressure inside the vacuum chamber occurring during the laser irradiation and indicative of the ablation of the target) to meet a good ablation rate and no substrate damage. These values of laser fluence are well consistent with IMAPLE deposition condition [58]. All samples underwent irradiation with about 3000 laser pulses. The depositions were performed at room temperature on KBr pellets (13 mm diameter) for FTIR inspections and on home-made silicone (PDMS) slices (1 cm^2^, Figure S2 in Supporting Information) to verify the quality of the deposition on a typical biomedical substrate and for in vitro toxicity assessment.

### 2.5. Biological Studies

#### 2.5.1. In Vitro Toxicity Assessment

NIH3T3 murine embryonic fibroblast (ATCC, CRL-1658), HeLa human cervical adenocarcinoma cell line (ATCC, CRM-CCL-2) and HaCAT human immortalized keratinocytes (Life Technologies, Monza, Italy) were cultured in Dulbecco’s Modified Eagle Medium, (DMEM, Gibco^®^, Shanghai, China) supplemented with 10% fetal bovine serum (FBS, Life Technologies, Monza, Italy) 2 mM glutamine and 100 U/100 g/mL of penicillin-streptomycin (Gibco^®^) in a humidified environment with 5% CO_2_ and 95% atmosphere at 37 °C. For all the experiments, cells at passage 2 o 3 were used. For toxicity studies, the in vitro MTT assay was used. The assay measures the viability of living cells via the cleavage of MTT (3-[4,5-dimethylthiazol-2-yl]-2,5-diphenyltetrazolium bromide) to formazan crystals by the cells’ dehydrogenases activities. Cells were seeded at such a density that they do not reach the confluence in 48 h (6 × 10^3^ cells/well (NIH3T3), 5 × 10^3^ cells/well (HaCAT), 2 × 10^3^ cells/well (HeLa)) in a 96-well plate 24 h before exposure to the indicate concentrations of GL suspension. A set of each cell lines not exposed to GL was kept as negative control. The positive control was obtained from preliminary tests on the three cell lines treated with different concentrations of H_2_O_2_ and the latest concentration showing viable cells for each cell line was chosen for positive control of the assays. After 48 h of incubation at 37 °C in a humidified atmosphere of 5% CO_2_, 10 μL of the MTT solution (5 mg/mL in PBS,) was added to the 0.1 mL culture medium per well and the 96 well plates were incubated a 37 °C for another 4 h. The produced formazan crystals were then dissolved in 0.1 mL of the pre-warmed MTT Solubilization Solution (10% Triton X-100, 0.1 N HCl in anhydrous isopropanol). The absorbance of the formazan dye was measured in the Biotek Sinergy LX Multimode Reader equipped with the Gen5 software (Agilent, Santa Clara, CA, USA) at 570 nm and 690 nm. Cell viability was calculated as OD_570nm_ of sample/OD_570nm_ of control x100. Each measurement was performed in triplicate. The tested concentrations were considered not toxic if the relative cell viability is ≥70% of control cells, according with the criteria described in ISO-10993-5. For the direct contact test, the cells were seeded in a 96-well plate and cultured for 24 h. The PDMS and the PDMS covered by the GL film (hereinafter GL-MAPLE) samples were cut in small pieces (0.1 cm^2^, which cover about 1/3 of the well bottom area) sterilized by UV and added to the wells ensuring that the PDMS slices from the side of the coating was at close proximity to the cells monolayer but not pressed onto it. PDMS samples without coating were used as negative control. The cultures were incubated from 24 to 72 h, after that the pieces of PDMS and PDMS-GL slides were removed and the MTT assay was performed as above described.

#### 2.5.2. Light Microscopy

Phase–contrast light microscopy observations were performed in order to evaluate alterations in cell morphology after 48 h of exposure to GL suspensions and to evaluate the integrity of PDMS-GL slices removed from the cell culture medium after the various incubation times. Cells and PDMS-GL slices were visualized with a 10×/0.40 (dry lens) or 5×/0.30 objectives, respectively, using an inverted microscope (DMI4000, Leica Microsystems, Wetzlar, Germany) at room temperature in 1× PBS. The images were captured with a digital camera (DFC365 FX, Leica Microsystems) using LAS-AF 2.0 software (Leica Microsystems).

#### 2.5.3. Statistical Analysis

The number of biological replicates of each experiment is indicated in the figure legends (n). The means of at least 2 independent experiments were used to calculate standard error of mean (SEM) or standard deviation (SD) and to perform statistical analysis (when appropriate). All *p* values were calculated by Student’s *t* test.

## 3. Results and Discussion

### 3.1. Threshold Identification

In order to confirm the successful deposition of GL, a chemico-physical characterization of GL films deposited via MAPLE was performed by FTIR survey in transmission mode and by optical microscopy and AFM investigations. To this aim, the GL films were deposited on PDMS slices and also on the KBr pellet (hereinafter GL-MAPLE).

The deposition threshold of GL was assessed by testing three laser pulse energy values: 120 mJ/pulse, 230 mJ/pulse and 350 mJ/pulse. The maximum laser pulse energy obtainable with the laser used in the used experimental set-up (350 mJ/pulse, corresponding to a fluence of 25 J/cm^2^) was at first tested. FTIR survey and microscopy imaging (optical microscopy and AFM) confirmed that such value was appropriate to obtain a homogenous covering of the selected support (KBr pellet and PDMS slice, Figure S3, Supplementary Information). The lower threshold, 230 mJ/pulse, corresponding to F = 16 J/cm^2^, was then identified by lowering step by step the laser power. Below this F value there was no GL deposition detectable by FTIR investigation. Only a faint shadow indicative of a very incomplete covering of the surface was observable by optical microscopy, but still detectable by AFM. At the laser pulse energy of 120 mJ/pulse, namely with a fluence value below F = 10 J/cm^2^, the PDMS substrate did not show any presence of GL material, even under AFM investigation. This threshold value was in line with those reported by Steiner et al. (~50 J/cm^2^, [58]) in the case of IMAPLE, an aqueous suspension with a concentration of nanoparticles of 1–2 wt.%. This value depends of course on the optical properties of the nanoparticles that in turn are determined by their size, shape, composition, and surroundings.

The film depositions were then performed at 350 mJ/pulse, above the deposition threshold, with the aim to optimize the deposition timing. The uniformity and the quality of the GL-MAPLE films were controlled by optical investigation after deposition. Images of GL-MAPLE films acquired by digital camera and by a phase–contrast light microscopy are reported in Figure S3, in the Supporting Material section.

### 3.2. FTIR Analysis

**Figure 2:**
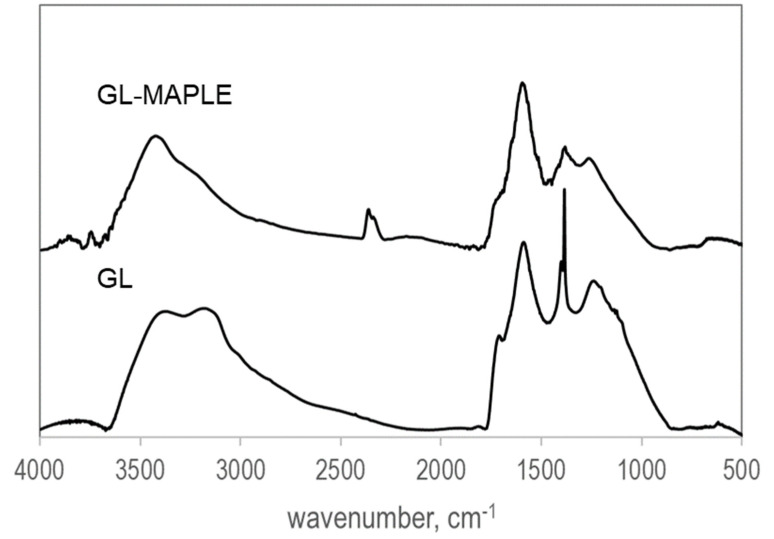
FTIR spectra of the GL and GL-MAPLE (laser pulse energy: 350 mJ/pulse) in the 500–4000 cm^−1^ region.

The FTIR spectrum of GL-MAPLE, acquired in transmittance mode, in the range 500–4000 cm^−1^, is contrasted with that of the parent GL in Figure 2. The spectra are baseline corrected and shifted for clarity.

The spectra are characterized by a broad shape, as typical exhibited by complex carbon networks [59]. The band around 2300 cm^−1^ is ascribable to CO_2_ in the environment and it arises as a consequence of the background subtraction. Bands attributable to oxygen functional groups (residual from the strong acid top-down demolition treatment) are slightly detectable [51,54]. Besides the enhanced broad band in the 1300 – 1100 cm^−1^ region ascribable to the overlapping of multiple sp^2^ graphitic skeletal stretching vibrations [59], the GL spectrum is typically poorly structured and presents a broad band in the 3000–3700 cm^−1^ range related to O-H stretching vibrations due to possible adsorbed H_2_O and, at lower wavenumbers, a broad N-H stretching band attributable to NH_2_ functionalities likely in the form of hydrazones) and a band at 1500–1600 cm^−1^ due to skeletal vibration of the sp^2^ graphitic domains. A weak shoulder at 1650–1750 cm^−1^ (C=O stretching vibrations) testifies the presence of residual carbonyl and carboxyl groups, anhydrides, lactones, single ketones and/or quinones. Nitrogen atoms in the form of nitro groups were also detected (typical -NO_2_ stretching vibration bands at 1560 and 1350 cm^−1^). The shape of the FTIR spectrum of GL-MAPLE does not present significant differences compared with the parent GL, indicating that the GL spectroscopic features upon laser deposition are overall preserved. It is noteworthy that the signal attributed to the -NO_2_ stretching vibration bands (around 1380 cm^−1^) is significantly reduced, as well as the broad N–H stretching band attributable to NH_2_ functionalities indicating a possible surface cleaning upon the laser ablation, suggesting the instauration of RIMAPLE phenomena involving the loss of the side functional groups [60]. During inverse matrix-assisted pulsed laser evaporation, photo-induced chemical reactions can occur and for this reason the technique is also known as reactive inverse matrix-assisted pulsed laser evaporation (RIMAPLE) [60]. GL is a thermally robust material (the decomposition of residual oxygen functional groups decorating the edge of the graphenic planes starts at about 200 °C and GL burn-off occurs at about 550 °C) [51], but the occurrence of RIMAPLE phenomena involving the loss of the decorating side functional groups is an added-value phenomenon since it can enhance the hydrophobicity of the coating originating by the GL landed on the substrate surface, thus enhancing its adhesion on PDMS. 

### 3.3. Surface Properties and AFM Imaging

**Figure 3:**
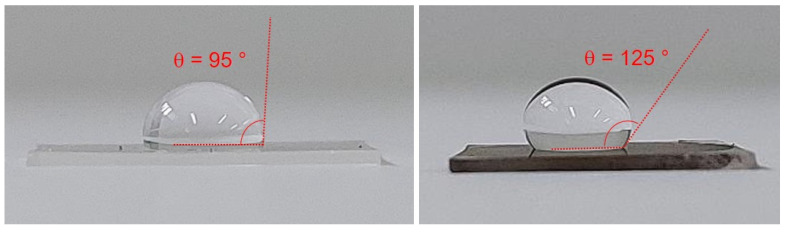
Contact angle values of GL-MAPLE film and neat PDMS surface.

GL is a hydrophilic material with a tendency to aggregate when dried due to the instauration of hydrophobic interactions between the hydrophobic basal planes [51,52]. The hydrophobic nature of GL-MAPLE films was demonstrated by contact angle measurements (Figure 3 and Figure S4 in Supporting Information). The higher value of the contact angle measured for GL-MAPLE film (θ = 125°) with respect to neat PDMS surface (θ = 95°) indicates that the GL-MAPLE film is clearly hydrophobic (90 < θ < 150). It is worth nothing that the increase of the contact angle could be also affected by the nanostructured features of the GL film, as detailed by AFM inspection reported in the following.

The morphology of GL before and after MAPLE deposition was inspected by AFM to evidence the occurrence of GL modification. To this aim, GL in water suspension was drop-casted on PDMS, dried, and directly inspected by AFM. Due to the hydrophobic nature of the PDMS, the dry GL in water suspension does not stick to the substrate surface (Supplementary Information, Figure S1), as expected. 

**Figure 4:**
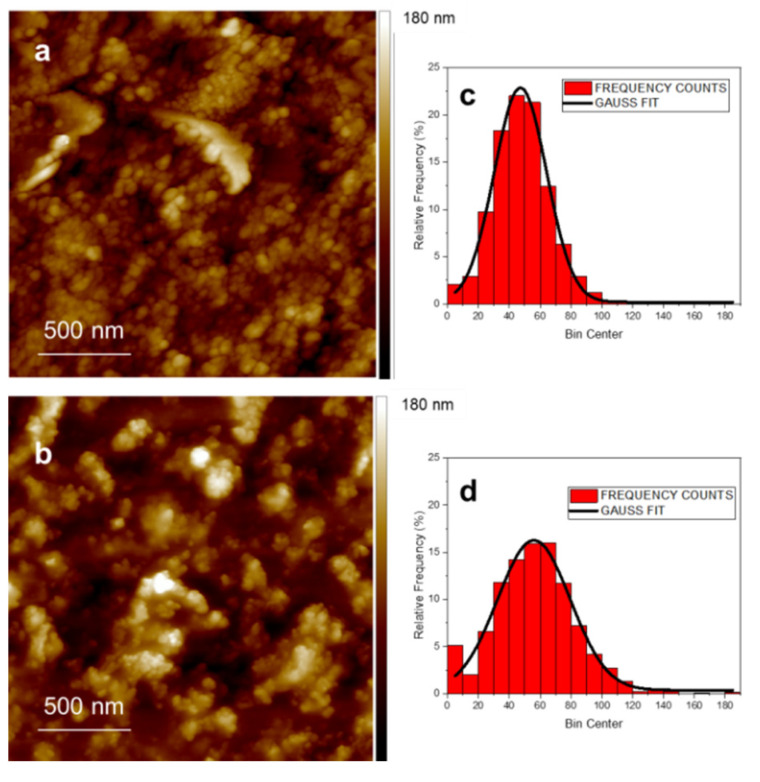
Non-contact representative AFM images (2 μm × 2 μm) of drop-casted (**a**) and MAPLE (**b**) films deposited on PDMS substrate; GL nanoparticle size distribution (**c**,**d**).

Figure 4 shows representative AFM images of GL drop-casted (panel a) and deposited as film by MAPLE (panel b) on PDMS substrate. The size distribution of the GL nanoparticles in the deposited film have been estimated using image analysis as well as the surface roughness parameters (mean roughness, Ra, and Root Mean Square, RMS). The silicone RMS was preliminary evaluated as 2.5 nm (Supplementary Information, Figure S5). The AFM inspection demonstrated that the MAPLE deposition was successful in completely covering the surface of the substrate without discontinuity. As a matter of fact, an enlargement of the film surface (Supplementary Information, Figure S6) evidenced that the PDMS surface is completely covered by the GL.

Figure 4 insets show representative size distributions of the GL obtained by AFM images of films produced by drop casting (Figure 4c) and MAPLE (Figure 4d). The size distribution is well described by a normal function (full line) in both cases. The mean radius D with the geometrical standard deviation σ is D = (47 ± 34) nm for the drop-casted sample, and D = (56 ± 47) nm for MAPLE film, respectively. The size distributions show similar characteristics for the two films and with those previously found in separately deposited nanoparticles [34]. GL nanoparticles stick to each other during film formation, while maintaining their own individuality. This suggests that the deposition (also by MAPLE) on a substrate held at room temperature significantly limits diffusion phenomena and coalescence processes in our experimental conditions. The surface roughness parameters (mean roughness, Ra, and Root Mean Square, RMS) were evaluated and the results were Ra = 17 ± 3 nm, RMS = 22 ± 5 nm for the drop-casted film and Ra = 21 ± 4 nm, RMS = 27 ± 6 nm for the GL-MAPLE film. These values are quite consistent although slightly lower surface roughness parameters are detectable for the drop-casted film. This circumstance is not surprising since in the case of MAPLE deposition, clusters of dry GL impacted the substrate without any settlement due to self-assembling phenomena arising upon slow drying when deposited in drop casting mode [52].

### 3.4. Biotoxicity Assessment

**Figure 5:**
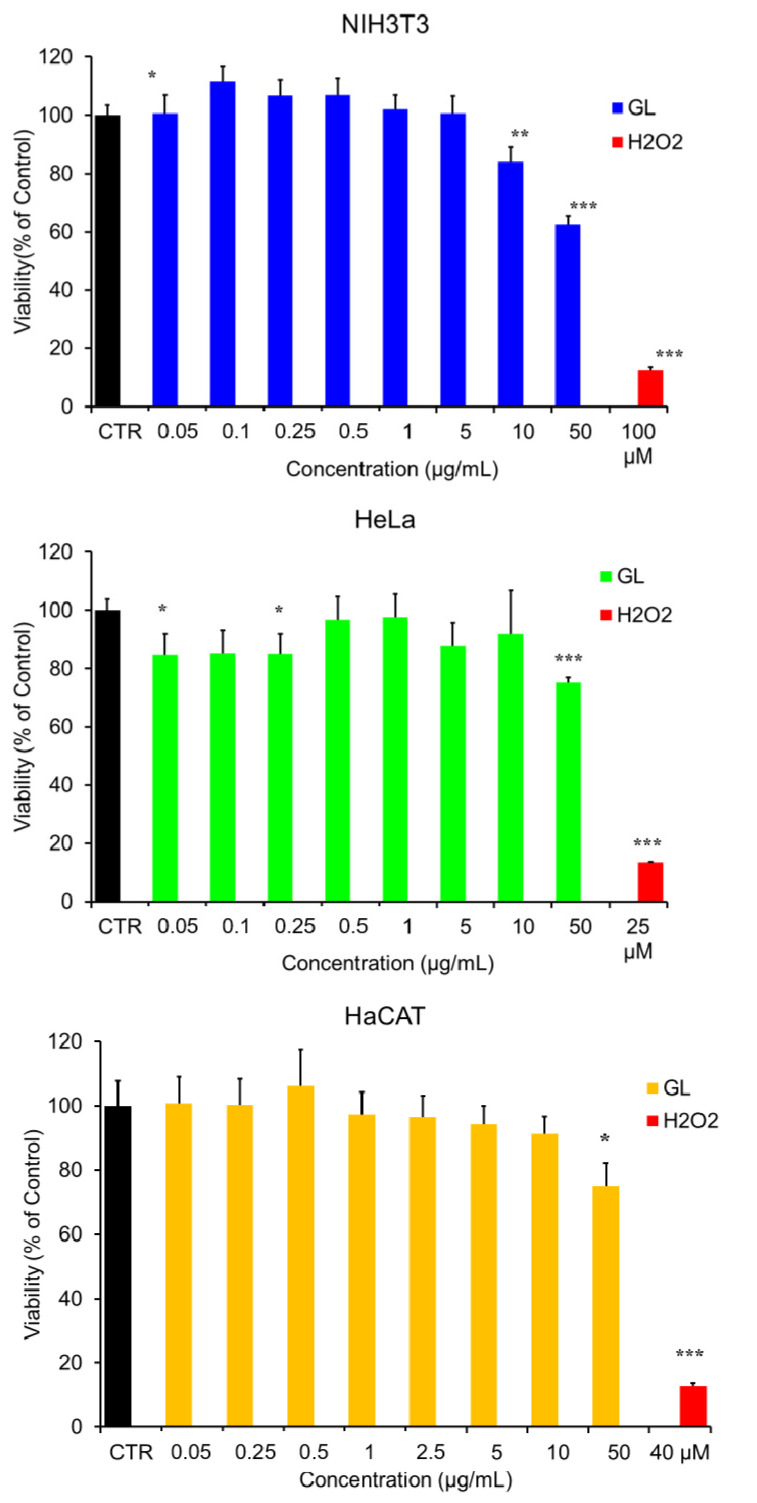
Cytotoxicity (percent of untreated control) of NH3T3 ((**upper**) panel), HaCAT ((**central**) panel) and HeLa ((**lower**) panel) cell lines determined by the MTT assay (±S.D.), following exposure to GL suspension at the indicate concentrations. Asterisks denote significant differences at *p* < 0.05 (*), *p* > 0.01 < 0.05 (**) and *p* < 0.01 (***).

The potential cytotoxicity of GL was investigated by the MTT [3-(4,5-dimethylthiazol-2-yl)-2,5-diphenyltetrazolium bromide] test (Figure 5), in order to evaluate possible interference in the cell mitochondrial function. 

The murine fibroblasts (NIH3T3), the human keratinocytes (HaCAT) and the human cervical adenocarcinoma epithelial-like (HeLa) cells were used to evaluate the response of different cell types following exposure. In particular, NIH3T3 cell line have been selected since a time-dependence and size-dependence cytotoxicity behavior towards graphene oxide (GO) have been found, differently from other adherent (A459, U87) and semi-adherent and suspension cells (RAW 264.7, NB4 and HL60) [61].

Before the MTT test, we verify the absence of side effects on cell growth and viability ascribable to neat PDMS slices. To this aim, the most sensitive cell line, NIH3T3, was exposed to direct contact with the PDMS slices for up to 72 hrs. As shown by the growth curves reported in the supporting material section (panel A, Figure S7), PDMS did not affect the rate of cells proliferation. The absence of cytotoxicity was also confirmed by the viability assay which showed no significant differences due to PDMS exposure (panel B, Figure S7).

Preliminary tests were conducted on all the three cells lines on GL water suspension at concentration ranging from 0.05 to 50 μg/mL for 48 h.

The MTT assay showed that GL did not result in any significant effect on the viability of the three cell lines (Figure 5) when it was used at concentration up to 10 μg/mL. Indeed, the decrease in cell viability was in any case less than 30% with respect to the control untreated cells. A slight effect of GL was detected only in NH3T3 (62% of viable cells) with the increase in concentration to 50 μg/mL. The trend towards the measured viability of cells and the increasing concentrations of the GL suspension is obviously dose-dependent. Moreover, one can notice that low doses of GL enhanced in most cases the viability of the cells.

**Figure 6:**
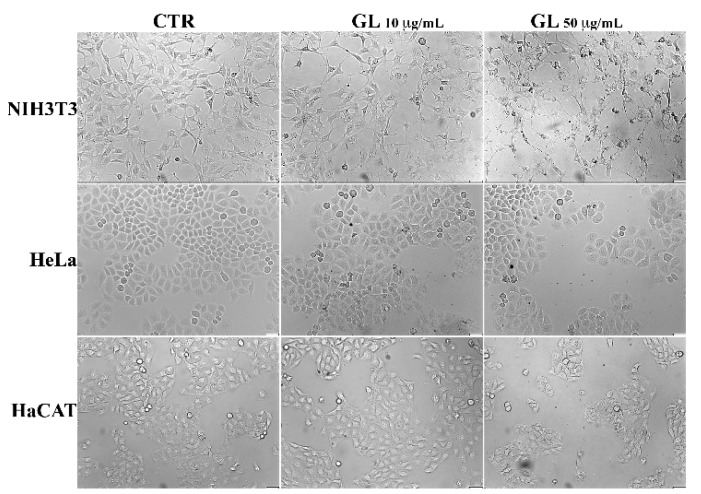
Representative images of morphology of cell monolayers untreated (CTR), ((**left**) panels) compared to cells treated for 48 h with GL at 10 ((**central**) panels) and 50 μg/mL ((**right**) panels). Scale bar 50 μm.

To further explore cytotoxicity of GL, we examined cultured cells by phase-contrast microscopy. Figure 6 shows the representative images of the morphology of untreated cell monolayers (CTR) compared to cells that have been treated for 48 h with GL suspensions at the concentrations of 10 and 50 μg/mL.

The analysis did not reveal differences between the morphology of GL-treated cells and the corresponding control cells. The vast majority of the cells were visually viable and remained spread on the surface of the wells; The HaCAT and HeLa cell populations include a low percentage of rounded cells, which is characteristic of these cultures. Furthermore, poorly attached or detached cells were not observed but the density of cells was lower than that of the control, indicating that GL did not have toxic effects even at high concentration, but it rather affected the cell proliferation rate.

**Figure 7:**
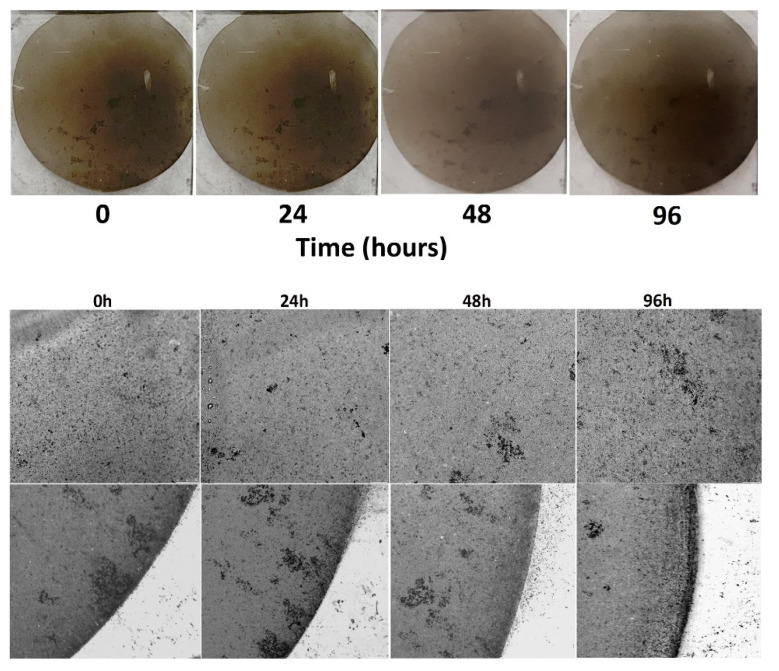
Representative digital photos of PDMS slices coated with GL-MAPLE and incubated in cell culture medium (DMEM) for 24–48 and 96 h at 37 °C with shacking. Images of GL-MAPLE films observed by phase–contrast light microscopy before and after the incubation. Images visualized with a 10×/0.40 ((**upper**) panels) or 5×/0.30 ((**lower**) panels) objectives, respectively. ((**lower**) panels) highlight a particular of the edges of deposit.

Before proceed with cytotoxicity testing on GL-MAPLE films, the stability of GL-MAPLE deposits in aqueous medium was probed. To this aim, GL-MAPLE films were incubated in the cell culture medium (DMEM) for 24, 48 and 96 h at 37 °C with shacking. The same slice was photographed before (0 h) and after 24, 48 and 96 h of incubation in the cell culture medium. Digital photos and phase–contrast light microscopy images of PDMS slices coated with GL-MAPLE before and after the incubation are reported in Figure 7. Results indicate a good resistance of the film over time in cell culture medium.

This result is particularly relevant as the GL-MAPLE films are expected to be used in the bladder at the contact with urine medium (average pH nearly neutrality and low percentage of inorganic salts, proteins, hormones, and a wide range of metabolites). 

**Figure 8:**
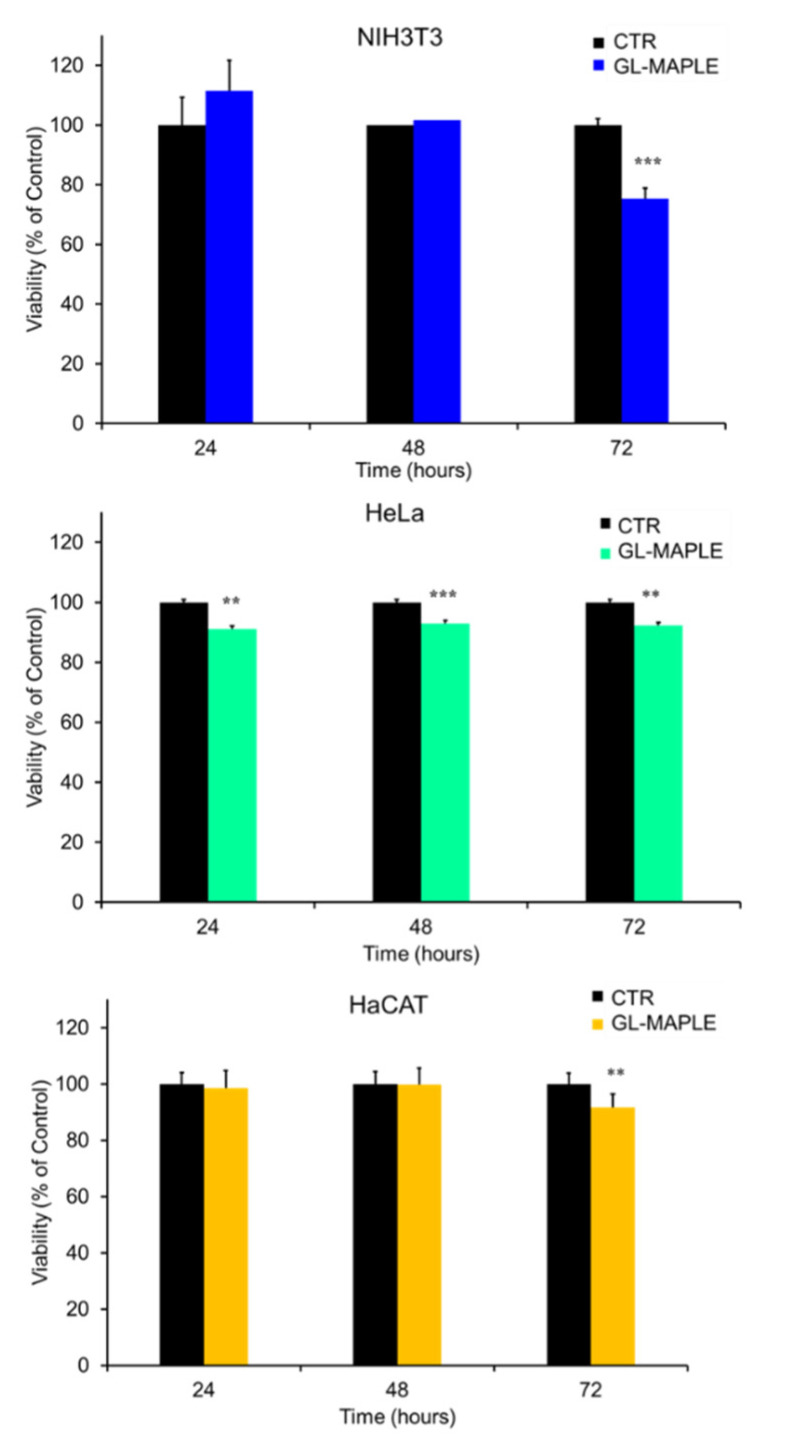
Cell viability measurement by MTT assay on NH3T3 ((**upper**) panel), HaCAT ((**central**) panel) and HeLa ((**lower**) panel) cell lines placed at direct contact with GL-MAPLE slices for the indicated time. Values are expressed as mean ± SD (*n* = 6). Asterisks denote significant differences at *p* < 0.05 (*), *p* > 0.01 < 0.05 (**) and *p* < 0.01 (***).

GL-MAPLE films on PDMS were tested for in vitro cytotoxicity by direct contact with cell monolayers. For MTT assay, GL-MAPLE slices were cut into small pieces covering about 30% of the surface area of cell monolayers and then cells were incubated for 24–48 and 72 h. The assay results reported in Figure 8 show that GL-MAPLE did not exhibit cytotoxicity in all three cell lines. 

The cell viability, indeed, was above 90% up to 72 h of incubation in HeLa and HaCAT cells and did not decrease under 75% in NIH3T3 fibroblasts.

Observation of the slices removed from the cell cultures with phase-contrast light microscopy showed the progressive detachment of small amounts of GL deposits from the PDMS support that were released in the culture medium (Figure S8, Supporting Information). Despite the observed leakage indicating a certain grade of instability of deposits, the released material showed a lower grade of toxicity with respect to free GL.

## 4. Conclusions

The progress in the MAPLE of graphenic materials obtained from CB has been investigated. The testing was performed on GL as a promising candidate for in vivo applications. GL deposited on PDMS substrates from water suspension via MAPLE were inspected by a biological survey toward target cellular lines (murine fibroblast NIH3T3, human keratinocytes HaCAT and the human cervical adenocarcinoma epithelial-like HeLa) demonstrating no perturbations in the different biological parameters evaluated, including the cytotoxic potential. GL, differently from most GRMs, are confirmed to be promising bio safe nanomaterials, stable in water suspensions and suitable to be firmly deposited by MAPLE approach in thin film on soft substrates. The GL films deposited by MAPLE exhibit a good resistance over time in cell culture medium. Specific tests to probe the resistance under different pH are planned in the future with the aim to expand the exploitation of the GL-MAPLE films in other market segments.

These results and the possibility to further functionalize the GL with suitable target molecules (e.g., contact killing molecules, slow release drugs) or combine them in hybrid materials [53] to assure a tighter adhesion to the substrate for use in harsh conditions, open the door to practical application of these new-concept devices for wide applications as biosensors and nanomedicine (drug delivery, next generation flexible devices, multifunctional coatings).

## Data Availability

Data is contained within the article and the Supporting Information.

## Supplementary Materials

The following supporting information can be downloaded at: https://www.mdpi.com/article/10.3390/nano12203663/s1, Figure S1. GL in water suspension drop-casted on PDMS substrate; Figure S2. PDMS substrate on the holder before and after the GL layers deposition; Figure S3. Images of GL-MAPLE films; Figure S4. Water drop (30 μL) on neat PDMS slice (left) and on GL-MAPLE film (right); Figure S5. AFM of PDMS substrate; Figure S6. AFM images GL-MAPLE; Figure S7. Effect of PDMS on NIH3T3 proliferation and viability; Figure S8. GL-MAPLE slides after incubation (0–72 h) on cell monolayers.
