# Supplementary material for: Coating of Flexible PDMS Substrates through Matrix-Assisted Pulsed Laser Evaporation (MAPLE) with a New-Concept Biocompatible Graphenic Material
Source: Nanomaterials (Basel). 2022 Oct 18;12(20):3663. doi: 10.3390/nano12203663 (PMC9610489; doi:10.3390/nano12203663)
Supplement: Supplementary file 1 [file nanomaterials-12-03663-s001.zip › nanomaterials-1931967-supplementary.pdf]

Supplementary information

Coating of flexible PDMS substrates through Matrix-Assisted Pulsed Laser Evaporation (MAPLE) with a new-concept biocompatible graphenic material

Michela Alfe,[*a] Giuseppina Minopoli,[b] Massimiliano Tartaglia,[b] Valentina Gargiulo,[a] Ugo Caruso,[c] Giovanni Piero Pepe,[d] Giovanni Ausanio[d]

[a] *Institute of Sciences and Technologies for Sustainable Energy and Mobility (CNR-STEMS), 80125 Naples, Italy.*
[b] *Department of Molecular Medicine and Medical Biotechnology, University of Naples Federico II, Naples, Via Pansini, 5, Naples, 80131, Italy.*
[c] *Department of Chemical Sciences, University of Naples Federico II, via Cinthia 4, 80126, Naples, Italy.*
[d] *Department of Physics "E. Pancini" University of Naples Federico II, via Cinthia 4, 80126, Naples, Italy.*

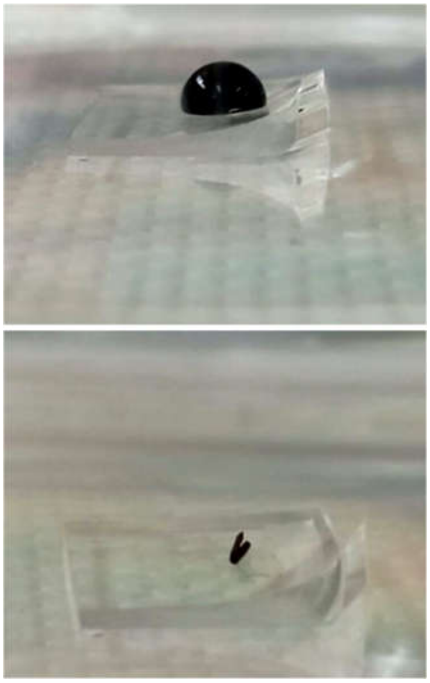

Figure S1. GL in water suspension (30 µl, 1% wt.) drop casted on PDMS substrate before (upper panel) and after drying at room temperature (lower panel).

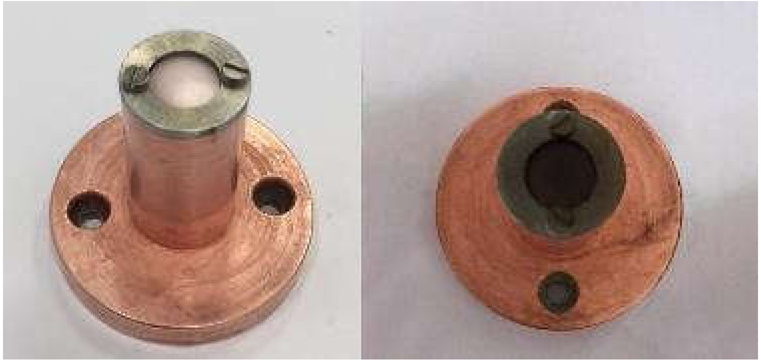

Figure S2. PDMS substrate on the holder before (left) and after (right) the GL layers deposition.

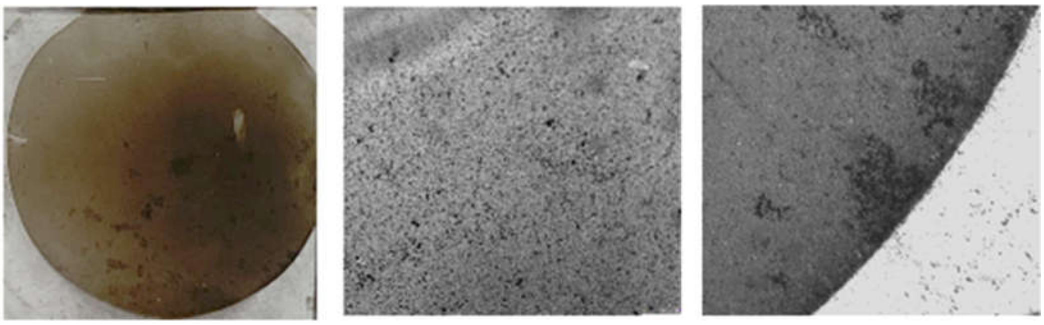

Figure S3. Images of GL-MAPLE films acquired by digital camera (left image) and by a phase–contrast light microscopy (center and right figures). Right figure highlights a particular of the deposit edges.

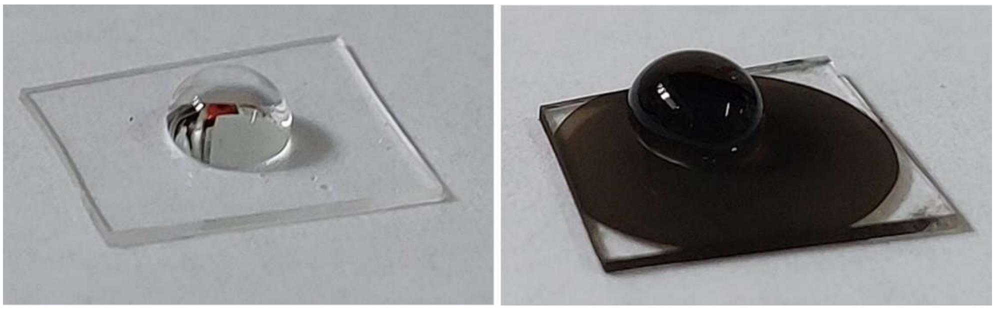

Figure S4. Water drop (30 μl) on neat PDMS slice (left) and on GL-MAPLE film (right).

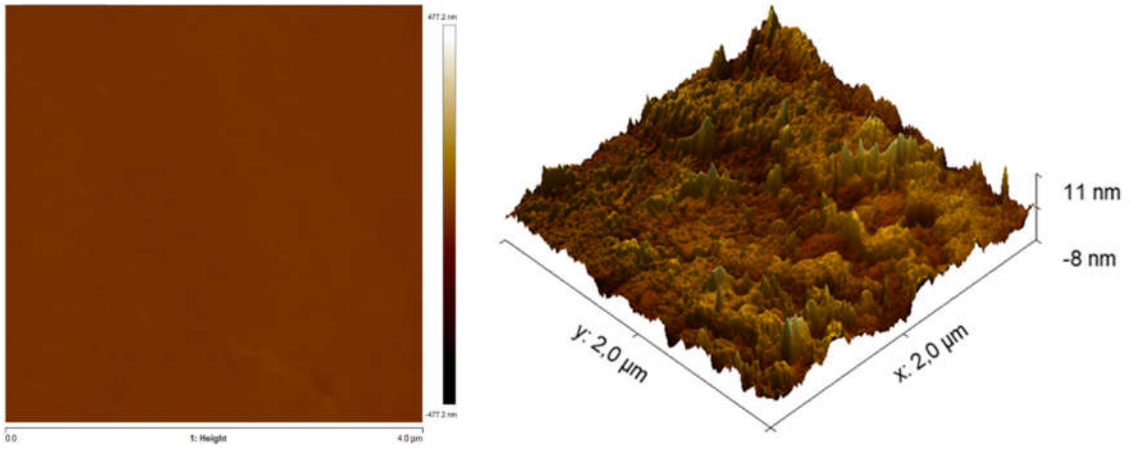

Figure S5. AFM of PDMS neat substrate, 4µmx4µm.

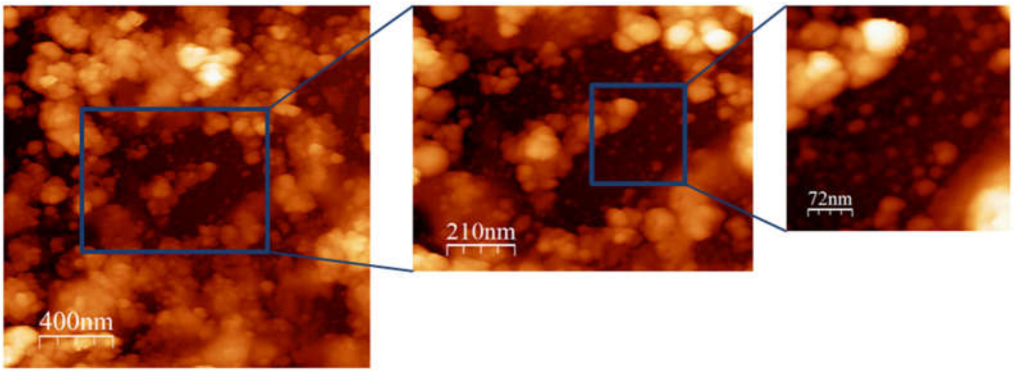

Figure S6. Non-contact representative AFM images (2 µm x 2 µm) of GL film deposited by MAPLE (350 mJ/pulse, fluence of 25 J/cm^2) on PDMS slides with enlargments, evidencing that the PDMS surface is completely covered by the GL.

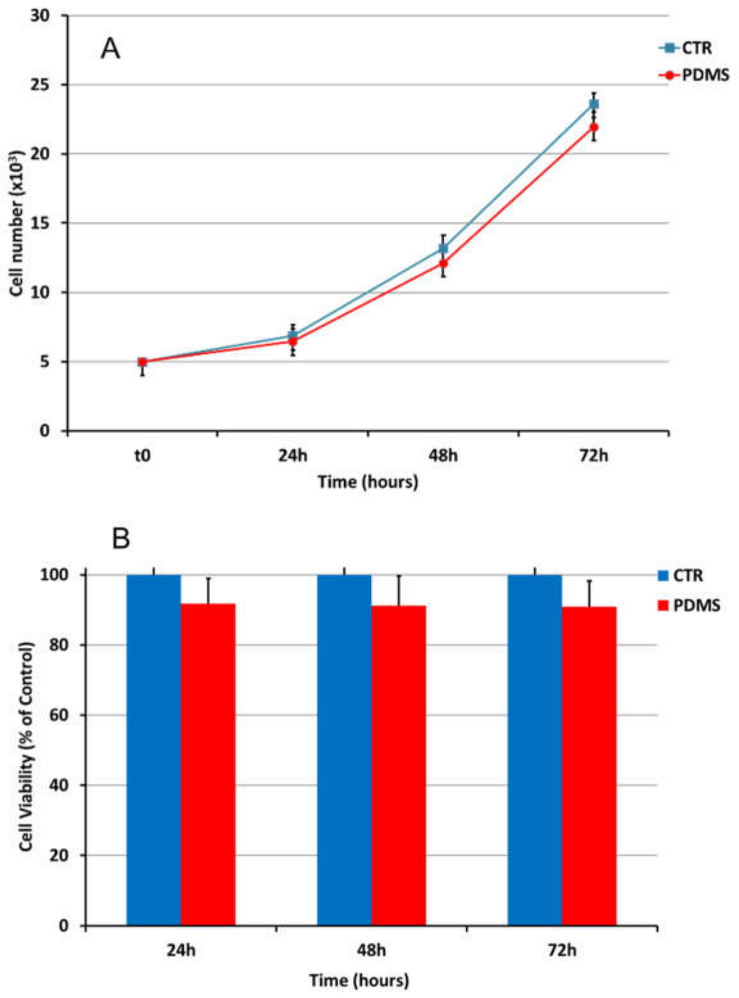

Figure S7. Effect of PDMS on NIH3T3 proliferation and viability. NIH3T3 cells were seeded at $5x10^3$ cells/well in 24-wells plate. 24 hours after cells were exposed to direct contact with PDMS slices. Cells number was counted after 24-48 and 72 hours (panel A). Cytotoxicity (percent of untreated control) of PDMS determined by the MTT assay on NIH3T3 cell line placed at direct contact with the slices for the indicated time (panel B). Values are expressed as mean ± SD (n=3).

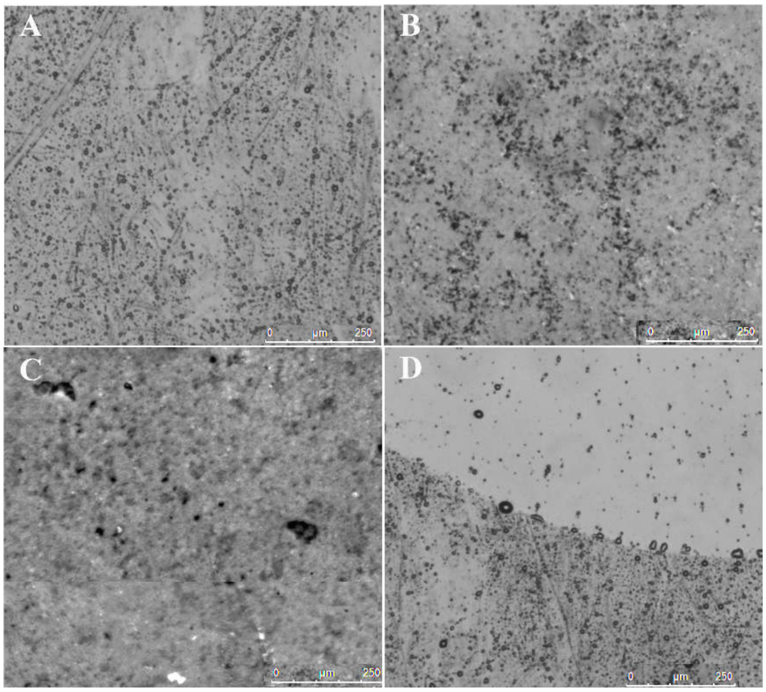

Figure S8. Representative micrographs of GL-MAPLE slides after incubation on cell monolayers for 24 hours (panel A), 48 hours (panel B) and 72 hours (panel C). Particular of a GL-MAPLE slide edge showing the release of deposited material in the medium (panel D). Scale bar 250 µm.
